# Improving end of life care in care homes; an evaluation of the six steps to success programme

**DOI:** 10.1186/s12904-016-0123-6

**Published:** 2016-06-03

**Authors:** Mary O’Brien, Jennifer Kirton, Katherine Knighting, Brenda Roe, Barbara Jack

**Affiliations:** Evidence-Based Practice Research Centre, Faculty of Health & Social Care, Edge Hill University, St Helens Road, Ormskirk, Lancashire, L39 4QP UK; Honorary Fellow, Personal Social Services Research Unit, University of Manchester, Manchester, UK

**Keywords:** Terminal care, End of life care, Qualitative research, Evaluation studies, Education, Palliative care, Advanced care planning

## Abstract

**Background:**

There are approximately 426,000 people residing within care homes in the UK. Residents often have complex trajectories of dying, which make it difficult for staff to manage their end-of-life care. There is growing recognition for the need to support care homes staff in the care of these residents with increased educational initiatives. One educational initiative is The *Six Steps to Success programme*.

**Method:**

In order to evaluate the implementation of *Six Steps* with the first cohort of care homes to complete the end-of-life programme in the North West of England., a pragmatic evaluation methodology was implemented in 2012–2013 using multiple methods of qualitative data collection; online questionnaire with facilitators (*n* = 16), interviews with facilitators (*n* = 9) and case studies of care homes that had completed the programme (*n* = 6). The evaluation explored the implementation approach and experiences of the programme facilitators and obtain a detailed account of the impact of Six Steps on individual care homes. Based upon the National Health Service (NHS) End of Life Care (EoLC) Programme, *The Route to Success in EoLC – Achieving Quality in Care Homes.*

**Results:**

The programme was flexibly designed so that it could be individually tailored to the geographical location and the individual cohort requirements. Facilitators provided comprehensive and flexible support to care homes.

Challenges to programme success were noted as; lack of time allocated to champions to devote to additional programme work, inappropriate staff selected as ‘Champions’ and staff sickness/high staff turnover presented challenges to embedding programme values.

Benefits to completing the programme were noted as; improvement in Advance Care Planning, improved staff communication/confidence when dealing with multi-disciplinary teams, improved end-of-life processes/documentation and increased staff confidence through acquisition of new knowledge and new processes.

**Conclusions:**

The findings suggested an overall positive impact from the programme. This flexibly designed programme continues to be dynamic, iteratively amended and improved which may affect the direct transferability of the results to future cohorts.

**Electronic supplementary material:**

The online version of this article (doi:10.1186/s12904-016-0123-6) contains supplementary material, which is available to authorized users.

## Background

There is growing recognition across Europe for the need to support the care of patients who have end-of-life needs within care home settings. This is endorsed in the UK End-of-life Care Strategy [1] and by the development of a dedicated taskforce by the European Association for Palliative Care (EAPC) [2]; central to its aims is the need to support the education of the care home workforce. The importance of early identification, discussion and documentation of end of life wishes by care staff and the need for all end of life care staff to have Advance Care Planning (ACP) training has recently been highlighted [3]. This sentiment is echoed in a recent report by Public Health England which noted that due to the increased number of deaths in care homes over recent years that more end of life care insight is required in the care homes sector [4].

It is estimated that by 2035 those aged 65 plus will account for 23 % of the total population, with those aged 85 plus expected to reach 3.5 million, or 5 %, of the total UK population [5]. An estimated 5,153 nursing homes and 12,525 residential care homes in the UK- often simply referred to as ‘care homes’, −have approximately 426,000 older or disabled people residing within them [6]. The national performance indicator ‘percentage of deaths in usual place of residence’ recognises that for many people, aged 65 plus, a care home is their usual residence. Although the proportion of deaths in care homes has increased over recent years, [7] internationally, deaths occurring in care homes are surprisingly low; in the UK, 18 % [8], in Austria 15 % and a higher rate of 39 % in Canada [9]. This is despite a majority of people [1] having a preference to die in their usual place of residence.

Residents are frequently admitted to care homes when no longer able to live independently; a situation likely to worsen due to the increasing prevalence of chronic diseases and significant growth in dementia rates amongst older people [10]. Multiple co-morbidities lead to complex end-of-life needs making it difficult and challenging for staff to recognise and manage the terminal phase [11]. Care homes face particular difficulties when providing EoLC; many residents may not experience a clearly identifiable ‘terminal illness’ making provision of appropriate care challenging [12]. Lack of support from GPs, a reluctance to prescribe appropriate medication and inconsistent availability of equipment, such as syringe drivers, are known to hinder care homes’ management of residents’ final illness [13] and their ability to prevent residents being hospitalised, at the end of their lives, who might otherwise be supported in the care home [14]. Public Health England [1] estimates that considerable financial savings could be made per death (399 GBP [503EUR/627USD]), for each death supported in the usual place of residence. Clearly an economic case exists, in addition to a strong compassionate one, for developing models of optimal EoLC for care homes.

Inappropriate admissions to hospital from care homes at EoL identified within the *End-of-life Care Strategy* [15] resulted in an appeal for more education for care home staff. It is believed that targeted training for care home staff will not only increase the palliative care capacity of staff, but can also potentially improve resident outcomes [16]. Little evidence of the effectiveness of current educational interventions in palliative care in care homes exists [17] and it has recently been documented that some staff do not have the skills and knowledge necessary to deliver good end-of-life care [18].

There is undoubtedly an urgent need for education in palliative care in care homes; staff describe themselves as lacking in confidence in providing EoLC and rate the extent of their EoLC training as ‘limited’ [19]. Recently there have been a number of educational initiatives in the UK designed to fill this gap in the EoLC education of care homes staff [20, 21]. One initiative is The *Six Steps to Success programme* (*Six Steps*) developed by a consortia of cancer and end-of-life care networks in the North West of England. The programme provides a flexible educational package and organisational change programme to help care home staff deliver the best EoLC. Based upon the National Health Service (NHS) EoLC Programme, *The Route to Success in EoLC – Achieving Quality in Care Homes* [19], the *Six Steps* programme has a workshop format addressing the core phases of EoLC within a six-stage cycle (see Table 1). The modular design of the *Six Steps* programme can be delivered flexibly to staff from individual homes or simultaneously to staff from several homes along an individual timeline, enabling it to be tailored to specific needs of diverse long term care facilities.

**Table 1:** Structure of the six steps programme

Structure of the six steps programme	
Step 1: Discussions as EoL approaches. Step 2: Assessment, care planning and review. Step 3: Co-ordination of care. Step 4: Delivery of high quality care. Step 5: Care in last days of life. Step 6: Care after death.	

In addition to delivering the workshops, a Facilitator will support the care home with the implementation of EoLC changes in the home and the preparation of the portfolio to demonstrate alterations to practice and impact of the programme on care. The *Six Steps* was undertaken in the North West of England and was commissioned by The Greater Manchester & Cheshire Cancer Network, the Merseyside & Cheshire Palliative and End of Life Care Network and the Cumbria & Lancashire End of Life Care Network and delivered by dedicated end-of-life care facilitators.

In addition, the programme incorporates mandatory education updates that include training on communication skills and ACP. Nominated care home staff (*Champions*) are the lead within the Care home for the *Six Steps* programme, they attend the workshops and cascade the information to all home staff. They are supported by an EoLC Facilitator who delivers the *Six Steps* workshops and provides tailored support and education to all staff. To successfully complete the programme care homes are required to maintain a portfolio containing evidence of how the care home has implemented *Six Steps*. Portfolios were developed as each step was delivered giving care homes time after the course had finished to complete them. Guidance and continual support was given to care homes to ensure they understood what should be included in the portfolio.

There is little evidence of the impact of educational initiatives on EoLC in the care home sector [20, 22]. This paper reports on an evaluation of the implementation of *Six Steps* with the first cohort of care homes to complete the programme; data were collected between September 2012 and January 2013.

### Aims

To explore the experiences of the facilitators of the programme, specifically with regard to the implementation approach they had adopted.

To obtain a detailed account of the impact of Six Steps on individual care homes.

## Methods

The independent evaluation, was undertaken in three geographical network areas in the North West of England. The design was tailored to meet the specific needs of the project reflecting the pragmatic stance to evaluation advocated by Rossi et al. [23]. Therefore in this study it was necessary to tailor the evaluation to consider the different time frames care homes were working to on the programme, the geographical coverage of the programme and the multiple elements to the programme including: audit data, wide variation in care homes and different models of course delivery implemented in addition to the differing needs of the stakeholders.

Furthermore, in keeping with this perspective, multiple methods of data collection were adopted [24] and care was taken to ensure that the purpose of the study was clear to all participants and that they were assured of anonymity. The independent evaluation consisted of two phases of data collection; qualitative data from facilitators through questionnaire and interviews; and case studies with a sub-sample of the care homes who participated in the programme. The research team were aware that facilitators and care homes may feel some pressure to be very positive about the programme and they tried to minimise the effect of this by making clear the purpose of the study, the anonymity of the participants and to inform them how the data would be handled directly by the University research team. A project advisory panel was formed to guide progress in the study, with regular meetings being held throughout the duration of the study. The project advisory panel comprised of representation from the three regional palliative and end of life networks, the research team and an independent NHS consultant. Regular meetings were therefore held with individuals who were familiar with the programme and the locality and were able to signpost and support the project team. In addition, all standard university quality reporting mechanisms were also adhered to including: a bi-monthly report to the research centre, quarterly report to the faculty research committee and report to the faculty ethics committee.

This paper reports on the qualitative data collected during the evaluation.

### Ethical considerations

The study was deemed to be service evaluation by NHS National Research Ethics Service, and the protocol was approved by the Faculty Research Ethics Committee from the host University and registered [LTC33]. Comprehensive information sheets were given to all participants in the research. Implied consent was given by participants that took part in the questionnaire and written informed consent was taken from each interviewee during the case studies.

### *Six steps* facilitators experiences

The aim was to explore the experiences of the facilitators of the programme, specifically with regard to the approach they had adopted. We were interested in what they judged had worked well, challenges faced and how they were overcome. An electronic survey with facilitators was employed as this was the optimal way to communicate with the sample population [25]. This was followed by individual interviews with facilitators to provide more depth to the findings [26].

A purposive approach to sampling was adopted [27], only those facilitators (*n* = 18) who had completed the programme with at least one care home were eligible for inclusion.

### Electronic questionnaire

The questionnaire was developed in consultation with the project advisory panel. The questionnaire asked about facilitators’ experiences of delivering *Six Steps,* and collected information on group demographics and model of delivery (see Additional file 1 for a copy of the questionnaire).

An email was sent to the eligible facilitators (*n* = 18) by the EOL leads from the three networks inviting them to participate in the study. The email contained an information sheet together with a link, via Survey Monkey® to the online questionnaire containing open and closed questions. It was emphasised several times that completed questionnaires were anonymous and would be accessed only by the research team; it was felt that this emphasis was important so that respondents felt able to be open and honest in their responses without risk of any impact on their role.

### Telephone interviews

A second email was sent to the eligible facilitators (*n* = 18) from the EOL network leads, inviting them to take part in an interview. Facilitators were asked to follow a link within the email to register their expression of interest in taking part in a telephone interview, to provide contact details and to give the research team permission to contact them.

The interview guide was developed in consultation with the project advisory panel and was informed by the data collected in the questionnaire phase of the evaluation (see Additional file 2 for a copy of the interview guide). Interviews focused on interviewees’ perceptions and experiences of their role as facilitator to the *Six Steps* and again the research team placed great emphasis on the fact that their responses would be anonymised so that their identify was protected.

### Care home case studies

The aim was to obtain a detailed account of the impact of *Six Steps* on individual care homes [28]. Care homes that had completed the programme in cohort one were invited to participate via contact from the EOL network leads. Thirty care homes volunteered to take part. Two care homes per network (*n* = 6) were independently selected as case studies by the research team using pre-determined selection criteria consisting of geographical location and Social Economic Status (SES) to facilitate the inclusion of a range of experiences from differing areas in the North West. For each case study, semi-structured interviews were held with the clinical lead, Champion, facilitator and clinical staff at each home; in addition the care home’s *Six Steps* portfolio was examined and mapped against the *Six Steps* evidence requirements. The research team were very clear about the exact purpose of the interviews so that their role was not misconstrued.

The portfolio was a folder comprised of a section for each of the six steps e.g. Section one focused on *Discussions as the end of life approaches* and contained subsections of the various elements. The portfolios had all been assessed by the Facilitators as part of the programme, with those not having the correct contents being required to improve. During the case studies we used the same check list as used by the Facilitators, but looked for ongoing examples of good practice and then explored its use by the staff – see the example in Table 2.

**Table 2:** Example of the portfolio content assessed for step 1 sections in the case studies

Step 1 - Discussions as the end of life approaches Not all care home residents are in the last year of life. The first step on the route to success is about identifying residents who are thought to be in their last year of life so that discussions around end of life care and advance care planning can be initiated.			
**1.1.**There is a policy or action plan developed within the care home for end of life care	**QM 5.1 Care Home Policy - End of Life Care** Should include all components of the Six Steps Programme. Copy of care homes Philosophy of Care	• Use of service users/families in the development • Any feedback from service users	**Case study factors noted;** • Is it a working document? – i.e. when reviewed/discussed
**1.2.**There is a system in place for identifying residents in the last year of life	**QM 5.3 End of Life Care Register** Photocopy of the populated register used in practice with patient details anonymised Evidence of a team approach to the register – could include minutes of meeting (including MDT) Documentation in residents notes Use of the North West Tool	Operating procedures/policies for identifying -Patients in last year of life -ACP, LCP, GSF, Syringe drivers -Spiritual assessment. Verification of death	**Case study factors noted;** Good practice : date of policy, who is to review it, How do we know staff have see them (Register of staff) End of life registers – evidence of review for ACP Patient records & communication with family

### Data analysis

Qualitative data collected through questionnaire and interviews were subjected to thematic analysis [26, 29]. To assure rigour qualitative data were analysed independently by two researchers (JK and BJ), by reading transcripts and listing emergent themes, before consultation to arrive at a consensus. QSR NVIVO 10 computer software was used to assist in this process.

## Results

### Facilitator participants

Sixteen facilitators (89 %) responded to the email invitation (*n* = 18) and completed the questionnaire. Nine facilitators subsequently volunteered to be interviewed giving a 50 % response rate. Facilitators had been in post for an average of 12 months, with a range of 3 to 18 months. They were mainly band 6 and 7 with one at band 8. Within the UK the pay banding system has registered staff on bands 5–8 and band 9 for senior managers with each band containing a number of pay points. They facilitated the programme in nursing, care and dual registered homes.

The findings from the questionnaires and interviews with facilitators are presented as a single set of results.

The themes reported are representative of the consensus of opinion evident and are illustrated with verbatim quotations labelled with their professional role and method of data collection (Interview (I) or questionnaire (Q) and sequential number as an identifier (e.g. facilitator QR4).

### Model of delivery

As expected with this flexibly designed programme there were marked differences in the way in which the facilitators chose to deliver the workshops, which was individualised to local need. Some facilitators delivered the programme from a central location, such as their local hospice. In areas with a wider geographical spread of homes, chose to deliver the sessions from different homes or venues each time; they felt this gave participants the opportunity to view other models of working and benchmark their practice against that of others.

Staff who attended the programmes as ‘Champions’ were reported to be mainly senior care staff or managers, with some junior care staff also attending. Facilitators felt that having a consistent Champion throughout the programme was extremely important to the success of the programme.

*‘The care home Champion is a vital link between the Facilitator and the home, the more senior the Champion the better when cascading the learning’* (Facilitator QR13).

The facilitators judged the documentation provided to them to guide delivery of *Six Steps* (such as the programme guide and information pack) as comprehensive and self-explanatory.

*‘It is really well structured - it followed the six step model from the EoLC strategy, so it's all very clear’* (Facilitator IR6).

### Support provided for homes throughout the programme

Facilitators provided flexible support to homes which was individually tailored to the need of each home. It generally consisted of a one to one visit to each home between each step to provide support. All facilitators also provided additional support and documentation via telephone and email throughout the programme, along with personalised support to assist the care home in building their portfolio.

### Ensuring commitment by the care homes

Many facilitators reported that it was extremely important to provide a very clear outline of the commitment required from care homes in order to complete the programme. This was in terms of time allocated by managers for staff to complete the additional work needed and a requirement of attendance at the face to face sessions.

*‘I’ve met with the manager and said to her that I didn’t think that the support was there for the staff … if she wanted her home to complete this program, she had a part to play in it as well’.* (Facilitator IR3)

### Challenges to care homes completing the programme

Facilitators reported that high sickness rates meant Champions could not always be released from the care home to attend training workshops. Furthermore staff turnover, within some homes, affected continuity. Challenges were also faced by facilitators when inappropriate staff were selected by the home to attend i.e. too junior to disseminate learning or implement change.

*‘Cascading information…they were really struggling with … I think it was the personality of the Champion, she was a very quiet girl and didn’t challenge anybody or anything,* (Facilitator IR3).

Facilitators reported that time was perhaps the biggest challenge to completion of the programme. Often staff were not given the time they were originally promised by managers, or they had underestimated the amount of work involved in the programme. Facilitators often acted in a mediator capacity with management in an attempt to negotiate time and access to electronic resources. Care home staff often reported doing work for the programme in their own time or on their days off, for example one respondent noted;

*‘… facilitation becomes very important because you go back to the manager and say ‘well when you started this programme you did say you would let the staff have a day, you did say that… you’d let them use a computer, you did say that you’d help with the printing’ and it’s feeding that back* (Facilitator IR2).

### Advance care planning (ACP)

According to one facilitator, staff on the programme grew in confidence once they had implemented, and experienced successes in ACP conversations;

*‘The most positive factor for me is empowering the care home Champions to draw up ACPs that result in far better communication and enable residents and families to think about wishes and preferences. Because these are documented everybody is aware and works towards fulfilling these requests’* (Facilitator QR4).

### Portfolio creation

Facilitators commented that the development of the portfolio was a challenging and very time consuming aspect of the programme;

*‘This was challenging for homes as they often had never done anything like this before. They needed support to understand the breadth of evidence that could be submitted’* (Facilitator QR6).

Some facilitators found that it was useful to formatively assess the portfolios prior to the end of the course which provided an opportunity to highlight any problems and provide additional support where if needed.

Care homes that had recently been inspected by external Quality Assurance (QA) agencies such as Care Quality Commission (CQC) reported an additional benefit of having a comprehensive portfolio to show inspectors.

*‘…a couple of homes had had CQC visits very soon after the programme… and they used the portfolio and the feedback we’ve given as part of the assessment visit…it’s great to have things there ready’* (facilitator IR1)

### Benefits to the home completing the programme

All facilitators reported that the care home staff who had completed *Six Steps* had experienced real benefits from the knowledge, skills and confidence they had acquired as a result. It was felt that the personalised support care home staff had received in working through the programme had helped them to change practice as one facilitator noted;

*‘One girl said the best thing for her was having someone to come in, help them to review what they’re doing and to change their practice because I think they felt it was quite hard to do that alone…’* (Facilitator IR1).

Facilitators reported that all homes judged their practice had improved as a result of being on *Six Steps*; furthermore some health professionals had also identified that there had been improvements within the care homes, as reported by this facilitator;

*‘They [care homes] all have reported that they’ve improved what they do and we’ve had feedback from some external professionals, just saying that … the homes were much more clued up on what was expected of them’* (Facilitator IR3)

One major factor that facilitators believed had been beneficial for the homes was that the staff had grown in confidence;

*‘It’s built their confidence so much it was unbelievable from when they started, …they even feel confident enough to challenge GPs’,* (Facilitator IR5).

No set protocol had been developed to support care homes once they had completed the programme. Most intended to keep in touch with care homes to offer additional support and check on progress. Some facilitators had assisted in creating care home forums to assist in sustaining the programme principles and to encourage peer support from other homes that had completed the programme. Workforce turnover was viewed as a major challenge to sustainability. Facilitators felt that a mechanism to support staffing changes and ensure that the *Six Steps* embedded values remained at the forefront of the minds of care home staff was needed; one facilitator summed up the thoughts of others:

*‘An induction programme for new starters across the cohorts could be developed in order to continue to embed and sustain the programme’* (Facilitator QR8).

Fundamental to the future of the programme was the need to provide ongoing support for the care homes; concerns were expressed by some facilitators about how this could be managed if funding for the programme was dis-continued.

*‘The process doesn't stop once the education has finished- it's the start of a big change and homes can require a lot of support through the process’* (Facilitator QR3).

### Case study participants

Six care homes were selected for the case studies using the pre-determined selection criteria outlined earlier resulting in 3 home from urban settings and 3 from rural settings (Table 3).

**Table 3:** Care home characteristics

	Registered	No. of beds
Case study 1	Residential home	34
Case study 2	Dual (nursing & residential)	53
Case study 3	Nursing home	33
Case study 4	Residential home	40
Case study 5	Nursing home	38
Case study 6	Residential (Dementia)	30

The findings from the interviews with staff and examination of the portfolios is summarised below.

### Summary of case studies

The care homes that were involved in the case studies were in the main very positive about the experience of attending *Six Steps.* There was consensus amongst care home staff that support provided for homes by the facilitators was broad and flexible and delivered according to individual care home need.

Homes reported a raised profile of EoLC within the home and significant improvements in their practice as a direct result of attending the programme. Staff confidence had increased through acquisition of new knowledge and new processes as highlighted by one care home manager;

‘*We were doing a lot of it already, … place but it formalised some things, some things we didn’t have in place, and it made us think about how we were doing things and actually how maybe to record things a bit better …’* (CS2, Manager).

As a result of participating in *Six Steps* ACP was now undertaken routinely with residents/families;

*‘… [ACP has] definitely improved, they’re finding it easier now to have conversations around ACP and all those other difficult conversations they have, they say they’re just a little bit easier now’* (Facilitator respondent IR1)’

Communication had improved with Multi-disciplinary Teams (MDTs). Care home staff reported feeling empowered to insist on the EoLC that they believed was correct for the resident. Staff described themselves as being more confident to challenge decisions of other professionals where necessary.

*‘… years ago, if a GP said… she doesn’t need EoL drugs, you’d have taken their word for that, but now we question that and we’ll argue to make sure that that person is settled, comfortable … and has a good death’* (CS6, Manager & Champion).

There was a sense that EoLC records within the homes had improved. Staff had created/implemented new forms of documentation which enhanced communication with external professionals through having evidence to support their decision-making. One participant emphasised the potential impact on care at the end-of-life.

‘*We can now say; ‘hang on a minute we don’t want an ambulance, we have all the paper work in place’.., so it has prevented unnecessary hospital admissions ‘(*CS5, Matron).

However, it was identified that cascading of *Six Steps* information was not done consistently and staff interviewed, who had not personally attended the programme, seemed unclear about the programme. At times this resulted in minimal/superficial understanding of changes that were being implemented as a result of the *Six Steps*.

## Discussion

It is recognised that lack of confidence amongst care homes staff has hindered the implementation of EoLC tools in the care home setting [1]. Furthermore, there have been calls for improvements to EoLC within care homes, which are underpinned by education and training [30]. As such the findings from this evaluation provide insight into a positive way forward for education and training for EoLC in the care home sector. *Six Steps* EOL programme resulted in a reported improvement in processes for delivering EoLC in care homes. Attending *Six Steps* training appears to have equipped staff in care homes with increased skills and confidence to provide EoLC. Overall, there was a sense of greater awareness of EoL issues amongst care home staff and a confidence to initiate ACP and to challenge health professionals if necessary which is consistent with research evidence [29].

Increased level of communication with MDTs as a result of attending *Six Steps* suggests that the programme is going some way to raise MDTs’ confidence in the ability of care home staff to deliver EoLC and thus potentially lessening discriminative attitudes by health care professionals, which is known to contribute to the isolation of care homes [13].

It was evident that the process of cascading the course information to the wider staff group needed much work. However it is acknowledged that this seems to be an effective planned approach that would be suitable in the care homes setting as the training could be delivered to staff, such as care assistants on site, who it is reported often find attending training sessions much more difficult [18]. However, it is clear that further work was required to ensure the information is cascaded appropriately and the correct member of staff was selected to manage the cascading process. Facilitators felt this was a key matter that selection of the Champion should be appropriate to the skills required for the role. It is suggested that further exploration and consideration of alternative modes of cascading information should also be sought. This could include the use of technology with the development of apps for use on phones and tablets which are rapidly developing in health care settings and which undoubtedly requires further research.

The results suggest the *Six Steps* Programme to be a valuable approach to enhance the EoL education of staff based in the care home setting. With the increased life expectancy of the population it can be anticipated that a care home will be for many, their normal place of residence. Therefore to support care and to enable place of death to be in the care home, it is vital that staff are prepared for proving EoLC. Facilitators and care home staff regarded Six *Steps* as a comprehensive programme that did not require amendment. Facilitators reported that the freedom and flexibility of delivery allowed them to adapt the programme to suit their own geographical area and the needs of the attendees; this is undoubtedly a strength of the programme. It should be noted, however, that this could also potentially lead to inconsistencies in programme delivery and potential fluctuations in standards of delivery.

Overall the programme appears to be effective, a model replicable across different regions and flexible enough to allow adaption to meet the needs of each care home including staff being able to engage with the programme. *Six Steps* Programme is a method of training that can be suggested to be effective in enhancing the knowledge and confidence of staff in supporting EoLC and could be widely adopted. Further research of the impact of the Six Steps Programme that includes patient reported outcome measures, including symptom control, and family’s views of the care their loved one received, are undoubtedly required to assess the full impact of the programme.

### Limitations of evaluation

This evaluation covers the first cohort to complete the programme; lessons learnt from the implementation of the programme have been applied to subsequent cohorts potentially ironing out many of the difficulties experienced during the first cohort. However, it is acknowledged that this could also potentially affect the transferability of the findings of this evaluation as the flexible nature of the programme meant that no two care homes had exactly the same experience. It is also acknowledged that facilitators reported learning from the experience with cohort one and had plans to modify the programme in the future to make improvements for future cohorts, meaning the programme was constantly evolving and adapting. This should be taken in consideration.

The research team were also aware that participants may have felt under pressure to be positive about their experience and consequently not highlight any difficulties they experienced. It may be that, particularly in the care home case studies, that the researchers’ role might be misconstrued as part of an audit. Time was take to carefully explain the role of the researchers and purpose of the research in an attempt to minimise this effect. However it is acknowledged that although every step was taken to avoid any confusion that some participants may still have felt unable to speak totally freely about their experience and it should be considered as a limitation of the study.

This paper reports only on the qualitative data collected. The quantitative data collected during the study was self-reported data, collected on a number of different systems and by various staff which resulted in numerous discrepancies which affected the quality of the data. Consequently we felt it inappropriate to report it here.

In compliance with confidentiality requirements, facilitators were initially approached to take part in the evaluation by the EoLC network leads. It is not known whether this also may have had an impact on their willingness to participate.

Thirty care homes volunteered to take part in the case studies, from which six were purposively selected. It is not known how these may differ from the other homes that were not selected; therefore the findings must be interpreted with caution and these results cannot be generalised to the wider population [27].

Although limitations are noted when interpreting the findings, it should be recognised that this evaluation has provided valuable insight into the positive impact of *Six Steps* on EoLC within the care homes.

### Recommendations

The evaluation only obtained facilitator and staff views on the implementation and effects of *Six Steps*. It is important that residents’ and family members’ views are sought, hence there is a need for further research specifically seeking the opinions of residents and families.

## Conclusion

With an increasing ageing population it can be expected that the number of people who move into care homes will continue to rise. For many people these care settings then become their home and therefore support to enable them to be cared for, and to die, there if they so wish should be promoted. Therefore there is a need to ensure care home staff have the necessary training and support to deliver effective EoLC.

This evaluation of the North West of England’s *Six Steps* EOL programme suggests that this flexible and adaptable model of training for care home staff, is starting to improve EoLC in care homes. The qualitative data, has suggested an overall positive impact from the programme. Care home staff reported it had increased their personal confidence in having EoLC conversations, this will undoubtedly go some way to help residents in their care to experience a ‘good death’.

## Abbreviations

ACP, advanced care planning; CQC, quality care commission; Eol, end of life; EoLC, end of life care; QA, quality assurance; SES, social economic status; Six Steps, six steps to success programme.

## Additional files

Additional file 1: Six Steps Facilitators Questionnaire. (PDF 546 kb) Additional file 2: Interview Guide for Care Home Staff. (DOCX 14 kb)
