# Supplementary material for: Improving end of life care in care homes; an evaluation of the six steps to success programme
Source: BMC Palliat Care. 2016 Jun 3;15:53. doi: 10.1186/s12904-016-0123-6 (PMC4891879; doi:10.1186/s12904-016-0123-6)
Supplement: Additional file 1: — Six Steps Facilitators Questionnaire. (PDF 546 kb) [file 12904_2016_123_MOESM1_ESM.pdf]

1. Additional file 1 Six Steps Facilitators Questionnaire

Thank you for responding to the link to complete this survey.

This is an independent evaluation that is being conducted by the University.

The survey will ask you questions about your experience as a facilitator of the Six Steps Programme in order to understand what has worked well and where lessons can be learnt.

The survey consists of 8 different sections;

1. Demographic and Care Home Information
2. Implementation of the programme
3. Examples of what has worked well
4. Examples of challenges and resolution
5. Your views of the Six Steps Programme
6. End of Life policy and portfolio for the Six Steps programme
7. Sustainability after the programme has ended
8. Final Comments

Please answer the questions as fully as possible to help us understand your experience of the programme and its implementation with the homes that have completed the programme. If you have taken over the role from another facilitator please answer the questions just in relation to your experience as a facilitator.

We will anonymise all responses so that individual care homes and facilitators are not identified in the survey results.

NB: This questionnaire must be completed in one session. This may take up to 30 minutes. Therefore it is suggested that you ensure that you have all information to hand relating to the homes you have facilitated.
Many thanks

* 1. Please enter your randomly allocated study code in the box below.

[ ]

2. Demographic and Care Home Information

2. How long have you worked as the 6 Steps Programme Facilitator?

- ( ) 1-6 months
- ( ) 7-12 months
- ( ) more than 12 months

3. Please indicate your current pay band.

- ○ Band 5
- ○ Band 6
- ○ Band 7

Other (please specify)

4. Which geographic areas do you cover? (Select as many PCT areas as apply)

- ☐ Ashton Wigan and Leigh
- ☐ Bolton
- ☐ Bury
- ☐ Central and Eastern Cheshire
- ☐ Heywood Middleton and Rochdale
- ☐ Manchester
- ☐ Oldham
- ☐ Salford
- ☐ Stockport
- ☐ Tameside and Glossop
- ☐ Trafford
- ☐ Halton and St Helens
- ☐ Knowsley
- ☐ Liverpool
- ☐ Sefton
- ☐ Warrington
- ☐ Western Cheshire
- ☐ Wirral
- ☐ Blackpool
- ☐ Blackburn with Darwen
- ☐ Cumbria
- ☐ North Lancashire
- ☐ Central Lancashire
- ☐ East Lancashire

5. Please provide the number of each type of Care Home in your area who have completed the programme.

Nursing

Residential

Mixed use/dual registered

Other (Please state)

6. How many Care Homes have left the programme before completing? (If you do not know please state 'Not Known' in the box below).

7. What are the main reasons Care Homes give for leaving the programme?

8. Please add any additional comments here for this Section

3. Implementation of the programme

The following questions are regarding the implementation of the 6 Steps programme in your area (while you have been in post).

9. How was the training delivered?

- [ ] To individual Care Homes
- [ ] By bringing the Care Homes together

10. Was there involvement from any other organisations?

- ○ No
- ○ Hospices

Other (please specify)

11. Typically, over how many months did you deliver the main 8 workshops?

12. Have you delivered the LCP and ACP/communication skills workshops?

- ○ Yes
- ○ No

13. Was there any variation in the facilitation and support provided by you to the Care Homes in your areas?

- ○ Yes
- ○ No

If Yes, please tell us about the variation and give some examples.

14. Please tick the boxes to indicate the type of support that you made available to the homes during the training. (Please tick all that apply)

☐ Telephone ☐ Email ☐ Face-to- face (with care home visit)

Other (please specify)

15. Please tell us an example of a method of support that worked well.

16. Please tell us an example of a method of support that you felt was not as effective.

17. Please can you give details of any initial feedback from GPs and multidisciplinary teams around their experience with the programme?

(Please give an example of the feedback received and the professional it came from).

18. Please tell us some examples of the things that have worked well during the implementation of the programme in your area.

(For example, factors which have led to successful training, engaging the staff in the training or the continuity of Care Home representatives throughout the programme).

4. Examples of challenges and resolutions

19. Please share some examples of any challenges that you have experienced during the implementation of the programme and how they were resolved?

(For example, recruiting Care Homes to the programme, multi-disciplinary team engagement and support, or continuity of the champion).

20. Is there anything that you feel could have been done better or differently to implement the 6 Steps programme?

5. Your views of the 6 Step Programme

21. Please rate how well you think the 6 Steps programme meets the following aims:

	Not Met				Met Fully	N/A
Improved quality of care for people nearing the end of life	○	○	○	○	○	○

Comment

	Not Met				Met Fully	N/A
Improved quality of support and care offered to families and carers of residents nearing the end of life	○	○	○	○	○	○

	Not Met				Met Fully	N/A

Comment

Improved collaboration between care homes and GPs, primary care teams, specialists, social care and others	○	○	○	○	○	○

Comment

Reduced hospitalisation in the last stages of life, enabling more residents to die in their care homes	○	○	○	○	○	○

Comment

Increased use of the Liverpool Care Pathway (LCP) in the homes	○	○	○	○	○	○

Comment

Increased use of Advance Care Planning tools in the homes	○	○	○	○	○	○

Comment

Decrease the number of unplanned admissions	○	○	○	○	○	○

Comment

Increased the number of residents dying in their Preferred Place of Care (PPC)	○	○	○	○	○	○

Comment

Care homes facilitating rapid discharges from hospital to the care home	○	○	○	○	○	○

	Not Met				Met Fully	N/A

Comment

Increased staff knowledge, skills and confidence in end of life care

Comment

22. How important to the success of the programme for individual homes were:

	Not Important at all				Extremely Important	N/A
Having continuity of Facilitator during the programme						
Having a champion through the programme (from start to finish)						

Comment

Comment

23. Please tell us how the End of Life policy was typically developed by the Care Homes that have completed the programme in your area, including any different approaches taken.

(Please give examples of the different approaches taken).

24. Please tell us how the portfolio of evidence was typically developed by the Care Homes that have taken part in Cohort 1 in your area, including any different approaches taken.

25. How many Care Home portfolios required further action plans?

26. Please describe some examples of the further actions needed.

27. Please tell us how you think the programme will be sustained in the Care Homes where the programme has been completed?

(Please give us some examples of any different approaches).

28. What challenges (if any) are there to the programme being sustained by the Care Homes that have completed in your area?

29. If you think there are challenges, do you have any suggestions for how these challenges can be resolved?

30. If you have any other comments you would like to make about the 6 Steps Programme and being a facilitator please tell us here.
