# Supplementary material for: Improving end of life care in care homes; an evaluation of the six steps to success programme
Source: BMC Palliat Care. 2016 Jun 3;15:53. doi: 10.1186/s12904-016-0123-6 (PMC4891879; doi:10.1186/s12904-016-0123-6)
Supplement: Additional file 2: — Interview Guide for Care Home Staff. (DOCX 14 kb) [file 12904_2016_123_MOESM2_ESM.docx]

**Additional file 2 - Interview Guide for Care Home Staff**

**(To be adapted for Manager - Clinical Lead - Champion)**

Demographic/General Questions

1. Please tell me about your experience of the following aspects of the 6 Steps programme?
   1. training received
   2. support from facilitator,
   3. identification of the local champion for the home
   4. benefits to the home of completing the programme,
   5. examples of things that have gone well,
   6. challenges to the home of completing the programme
   7. Have they had good support from management?
   8. challenges to sustaining the programme in the home after completion
2. How was the EOL policy developed for your home?
3. How has the EOL policy been implemented in your home?
4. Have you seen improvements in the EOL care at your home since completing the programme?
5. How was the portfolio of evidence developed for your home?
6. Did the original portfolio have any action plans?
7. What is the value of the portfolio to the home and external agencies?
8. Is the portfolio static or does it have any updates added?
9. Is there anything that you feel could have been done better or differently to implement the 6 steps programme for your home?
10. Is there anything else that you would like to have seen included in the programme?
11. Any other comments you would like to make about the programme?

**Guide 2: For Facilitators**

1. Please tell me about your experience of the being a facilitator of the 6 Steps programme?
   1. Training provided for you as a facilitator or previous experience
   2. Support you have received from others to assist facilitation
   3. training provided
   4. did you deviate from the specified 6 steps model
   5. support you have provided to the homes,
   6. identification of the local champion for the homes
   7. benefits to the home of completing the programme,
   8. examples of things that have gone well,
   9. challenges to the home of completing the programme – including any feedback from GP and other multidisciplinary professionals
   10. challenges to sustaining the programme in the home after completion
2. How have the EOL policies been developed for the homes?
3. How has the EOL policy been implemented in the homes?
4. Have you heard of/seen - improvements in the EOL care at the homes since completing the programme and implementing the EOL policy?
5. How has the portfolio of evidence developed for the homes? *(any differences)*
6. Is there anything that you feel could have been done better or differently to implement the 6 steps programme?
7. Is there anything else that you would like to have seen included in the programme?
8. Any other comments you would like to make about the programme?
9. What are your experiences around GP and multidisciplinary team buy-in of the Six Steps programme?
10. Have you received any positive/negative feedback from care homes around the programme and the impact on the care of residents?
